# The Bryozoa collection of the Italian National Antarctic Museum, with an updated checklist from Terra Nova Bay, Ross Sea

**DOI:** 10.3897/zookeys.812.26964

**Published:** 2019-01-03

**Authors:** Matteo Cecchetto, Chiara Lombardi, Simonepietro Canese, Silvia Cocito, Piotr Kuklinski, Claudio Mazzoli, Stefano Schiaparelli

**Affiliations:** 1 Italian National Antarctic Museum (MNA), Section of Genoa, Genoa, Italy Italian National Antarctic Museum Genoa Italy; 2 Department of Earth, Environmental and Life Science (DISTAV), University of Genoa, Genoa, Italy Department of Earth, Environmental and Life Science Genoa Italy; 3 Marine Environment Research Center ENEA, 19032 Pozzuolo di Lerici (La Spezia), La Spezia, Italy Marine Environment Research Center ENEA La Spezia Italy; 4 Italian Institute for Environmental Protection and Research, ISPRA BIO-HBT Department, Rome, Italy Italian Institute for Environmental Protection and Research Rome Italy; 5 Institute of Oceanology, Polish Academy of Sciences, Ul. Powstancow Warszawy 55, Sopot 81-712, Poland Institute of Oceanology, Polish Academy of Sciences Sopot Poland; 6 Department of Geosciences, University of Padua, Padua, Italy University of Padua Padua Italy

**Keywords:** Antarctica, Bryozoa, checklist, MNA, new records, outreach, Ross Sea, Terra Nova Bay, 3D models

## Abstract

This study provides taxonomic and distributional data of bryozoan species from the Ross Sea area, mainly around Terra Nova Bay, based on specimens curated at the Italian National Antarctic Museum (MNA, Section of Genoa). Bryozoan specimens were collected at 75 different sampling stations in the Ross Sea and in the Magellan Strait, in a bathymetric range of 18–711 meters, during 13 expeditions of the Italian National Antarctic Research Program (PNRA) conducted between 1988 and 2014. A total of 282 MNA vouchers corresponding to 311 specimens and 127 morphospecies have been identified and included in the present dataset. 62% of the species were already reported for the Terra Nova Bay area, where most of the Italian samples come from, with a 35% of samples representing new records classified at the specific level, and 3% classified at the genus level. These new additions increase to 124 the total number of species known to occur in Terra Nova Bay. Four 3D-models of Antarctic bryozoans from the Ross Sea are also presented and will be released for research and educational purposes on the Museum website.

## Introduction

In the last 30 years, several Italian expeditions have been conducted in the Ross Sea leading to the publication of contributions on different taxonomic groups. Among the different phyla, Bryozoa was extensively studied until 2000 with papers reporting data on bryozoan species obtained during the first Italian Antarctic campaigns in Terra Nova Bay (i.e., 1988–1995) (Di Geronimo and Rosso 1990; Rosso 1990, 1991, 1992a, 1994; Rosso and Sanfilippo 2000).

**Figure 1.**
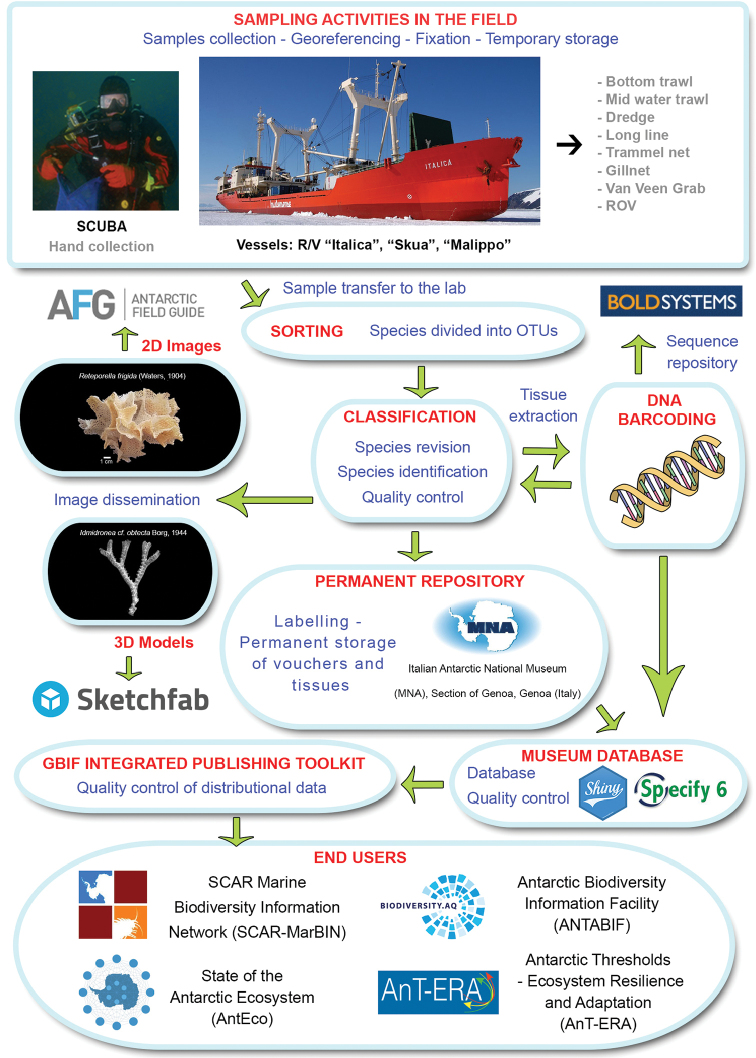
Flowchart depicting major stages in dataset development and publishing.

From 2000 onwards, new Antarctic campaigns were annually conducted and the number of new samples acquired by the Italian National Antarctic Museum (MNA, section of Genoa) progressively increased. However, no new taxonomic characterization was carried out on this material until now. Few samples from the Magellan Strait, collected during the VI Italian National Antarctic Program (PNRA) expedition in 1991, are also included here. The present study provides distributional data and taxonomic identification, at the lowest possible level, of bryozoans collected during 13 scientific expeditions of the PNRA in the Ross Sea and the Magellan Strait. These data are combined with previous literature checklists for the area with the aim of providing an updated checklist for Terra Nova Bay and distributional data for all the available vouchers. All bryozoan specimens reported in this paper are stored at the MNA and at the museum of the IPOP in Catania (Figure 1). This dataset is the sixth MNA contribution to the Antarctic Biodiversity Portal, the thematic Antarctic node for both the Ocean Biogeographic Information System (AntOBIS) and the Global Biodiversity Information Facility (ANTABIF) (http://www.biodiversity.aq). Previous contributions on Mollusca, Tanaidacea, Fungi, Ophiuroidea and Porifera were respectively published in Ghiglione et al. (2013), Piazza et al. (2014), Selbmann et al. (2015), Cecchetto et al. (2017) and Ghiglione et al. (2018).

## Project description

**Project title**: Antarctic Bryozoa in the collection of the Italian National Antarctic Museum (MNA)

**Curator and Promoter**: Stefano Schiaparelli

**Personnel**: Matteo Cecchetto, Chiara Lombardi, Simonepietro Canese, Silvia Cocito, Piotr Kuklinski, Claudio Mazzoli, Stefano Schiaparelli

**Funding**: The Bryozoa specimens were collected during 13 different Antarctic expeditions conducted from 1988 to 2014 and 18 research projects funded by the PNRA listed below (with the project name or category under the PNRA program in italic, followed by the project code, the expedition number and the corresponding years):

– Necton e risorse da pesca 2.1.4.6, III expedition (1987/1988)

– Oceanografia & Benthos 2.1.4.3, III expedition (1987/1988)

– Benthos 3.2.1.2.5, V expedition (1989/1990)

– Oceanografia geologica 3.2.1.4, V expedition (1989/1990)

– Benthos, Magellan strait 3.7.8, VI expedition (1990/1991)

– Ecologia e biogeochimica dell’Oceano Meridionale - ROSSMIZE 2d.2, XI expedition (1995/1996)

– Ecologia e biogeochimica dell’Oceano Meridionale 2b.3, XIII expedition (1997/1998)

– Struttura e dinamica delle cenosi marine di Baia Terra Nova 2b.3.1, XIV expedition (1998/1999)

– Processi genetici e significato paleoclimatico e paleoceanografico dei CARBONati marini biogenici in ANTartide - CARBONANT 4.7, XVII expedition (2001/2002)

– Molecole e geni di organismi marini antarctici in funzione evolutiva, adattativa e applicativa 1.1, XVIII (2002/2003)

– The costal ecosystem of Victoria Land coast: distribution and structure along the latitudinal gradient 2002/8.6, XIX expedition (2003/2004)

– L’ecosistema costiero di Baia Terra Nova - Latitudinal Gradient Project 2006/08.01, XXV expedition (2009/2010)

– Barcoding of Antarctic Marine Biodiversity - BAMBi 2010/A1.10, XXVII expedition (2011/2012)

– Vulnerabilità dei pesci polari al cambiamento climatico: ciclo vitale, habitats e relazione con il ghiaccio marino in Pleuragramma antarcticum 2010/A1.11, XXVIII expedition (2012/2013)

– Barcoding of Antarctic Marine Biodiversity - BAMBi 2010/A1.10, XXVIII expedition (2012/2013)

– Diversità genetica spazio temporale di endoparassiti delle regioni polari: uno studio per la valutazione dell’impatto dei cambiamenti globali sulle reti trofiche marine 2009/A1.09, XXVIII expedition (2012/2013)

– Integrità dell’ecosistema marino antarctico come presupposto per lo studio dell’interazione parassita-ospite: un approccio genetico, molecolare ed immunologico 2013/AZ1.09, XXIX expedition (2013/2014)

– Barcoding of Antarctic Marine Biodiversity - BAMBi 2010/A1.10, XXIX expedition (2013/2014)

**Figure 2.**
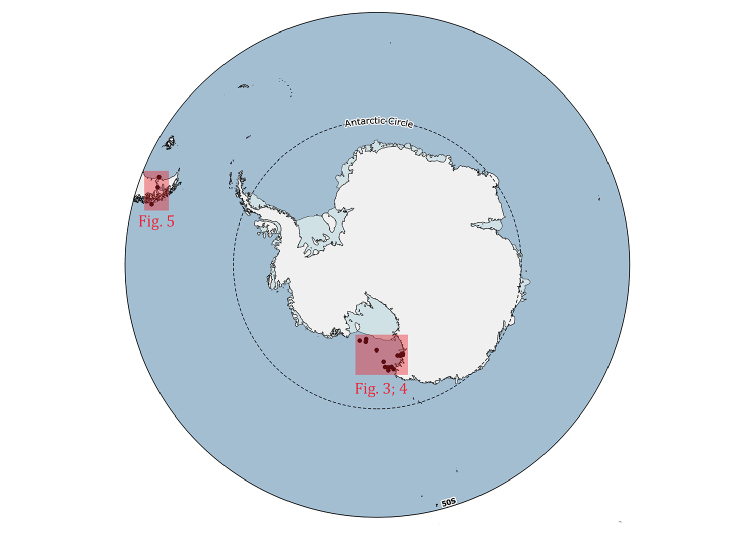
General map of Antarctica with the study areas highlighted (red boxes). Detailed maps of the sampling areas are provided in Figs 3–5.

**Figure 3.**
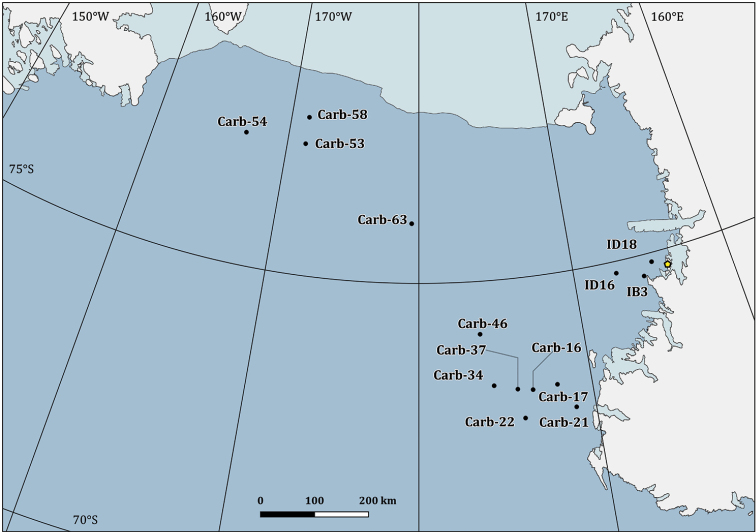
Sampling stations map in the Ross Sea area, Antarctica. The yellow pentagon indicates the location of the research station “Mario Zucchelli” in Terra Nova Bay. A detailed map of the sampling area for Terra Nova Bay is provided in Figure 4.

**Figure 4.**
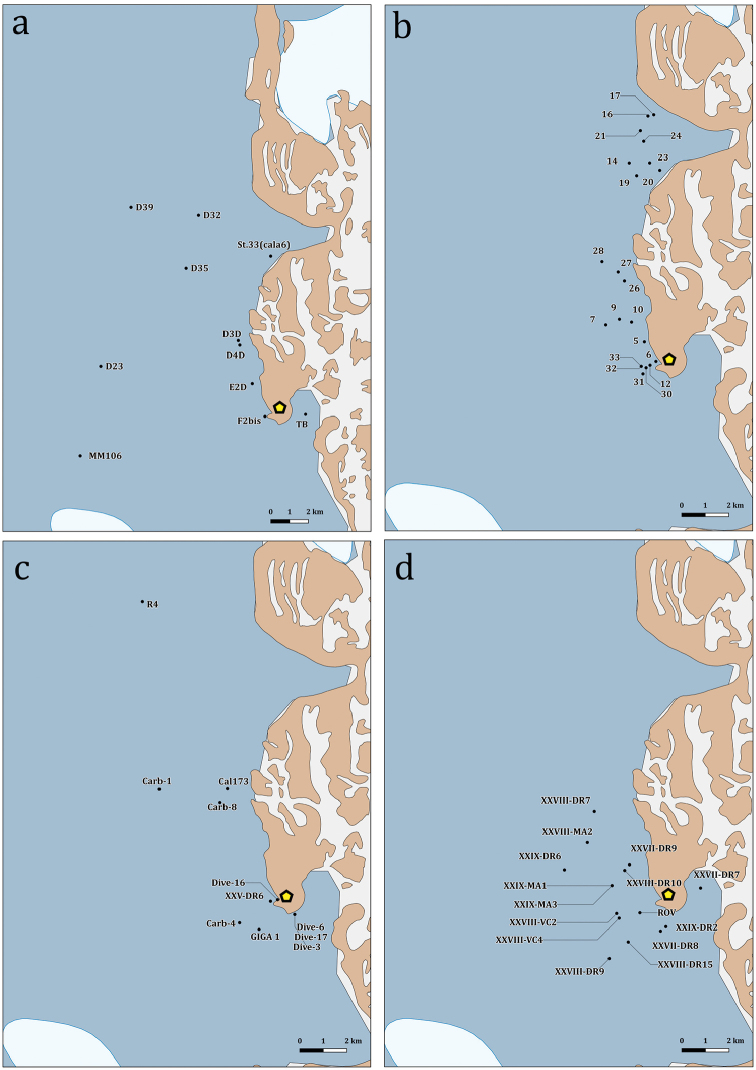
Detailed map of the sampling stations of the PNRA expeditions III, V and XI (**a**); XVIII (**b**); XIII, XIX, XVII and XXV (**c**); XXVII, XXVIII and XXIX (**d**). The yellow pentagon indicates the location of the research station “Mario Zucchelli”.

**Figure 5.**
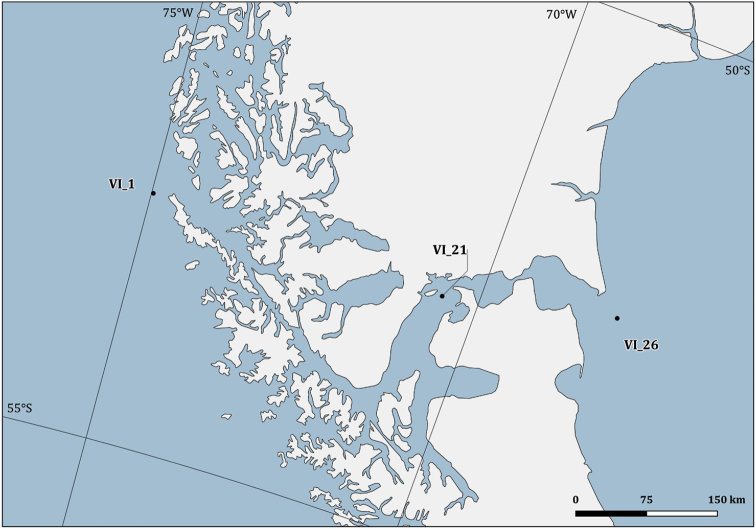
Detailed map of the sampling stations of the sixth PNRA expedition (Magellan Strait).

**Study area**: Bryozoa specimens were collected in the Ross Sea sector of the Southern Ocean (Figs 2–4) and in the Magellan Strait (Figs 2, 5).

**Design**: Data were assembled by revising all the distributional records of the specimens stored in the MNA collections (section of Genoa, Italy) and in the museum of the IPOP in Catania.

## Materials and methods

### Sampling

**Figure 6.**
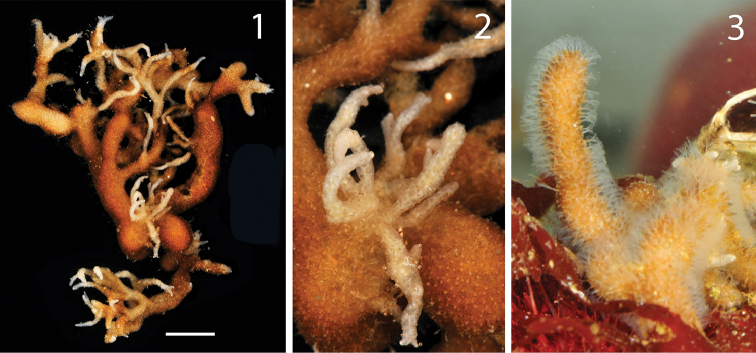
*Alcyonidium* sp. colony (MNA 2733) collected by SCUBA diving. Entire colony (**1**) and details (**2**) of the specimens after fixation in ethanol. Detail of the colony depicted in aquarium immediately after the collection (**3**).

**Figure 7.**
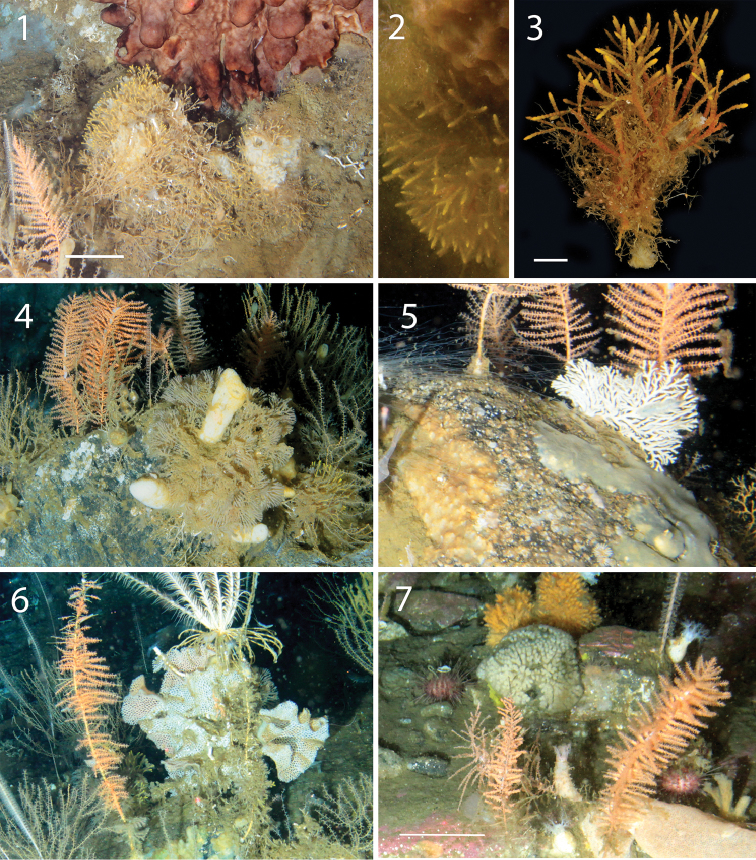
Images of bryozoan colonies in the field obtained through ROV video transect and SCUBA diving. Bushy-colony of *Klugellabuski* Hastings, 1943 (NR) living in association with other bryozoans, hydroids and sponge (**1**), a detail of the yellow tips of the colony in the field (**2**), and the whole fresh colony after the collection (**3**); the last two corresponding to the same voucher (MNA 2872) collected by SCUBA diving; colony of the cyclostome *Hornera* enclosing a sponge (**4**); fan-shaped colony of the cyclostome *Hornera* sp. growing on a boulder (**5**); colony of the cheilostome *Reteporella* sp. living in association with other bryozoans, hydroids, polychaetes (**6**); rounded greyish colony of the cyclostome *Fasciculiporaramosa* D’Orbigny 1839 growing on the sea bottom (**7**).

The distributional data of Bryozoa, here illustrated, came from different research expeditions, conducted between 1988 and 2014, and include 75 sampling stations, located between 18 and 711 metres of depth (Figs 2–5). Sampling was performed by using a variety of methods and gears such as dredges (Charcot dredge, Naturalist dredge, Triangular dredge, and Picard dredge), Van Veen grabs of different volumes and, for opportunistic sampling, fishing long lines, mid-water trawls (that accidentally touched the bottom due to a failure of the winches providing additional material), trammel nets and other fishing nets. In addition, during the XXV PNRA expedition, some samples were hand-collected by SCUBA diving by one of the authors (SS) (Figs 6, 7). Images of bryozoan colonies were also obtained through ROV video transects performed during the XXIX expedition, and subsequently identified at least to the genus level (Figure 7). Station coordinates and sampling events were recorded during sampling activities based on various GPS systems. The data flowchart (Figure 1) illustrates the sampling, sorting and storing procedures for specimens as well as data and image availability.

### Quality control

Once at the MNA, all specimens were classified at the lowest possible taxonomic level; only those that were classified at least to genus level were included in the present dataset. Several researchers contributed in classifying the specimens, and the last taxonomic revision of the MNA bryozoan collection was conducted in the past two years by Chiara Lombardi, Silvia Cocito, and Piotr Kuklinski. During all phases of sorting, classification and storage of samples at the MNA, quality control and data cleaning have been undertaken at various stages in order to produce high quality data, and make consistent cross-references between the database and samples’ labels. The MNA (www.mna.it) uses the R-Shiny web application (https://steu.shinyapps.io/MNA-generale/) to manage and show its collections, and a Microsoft SQL database (Specify 6) to link all the data (photos, glass slides, etc.) to the physical samples in the collection. Of a total of 282 MNA vouchers, 29 samples were collected and studied before the MNA was established in 1999, and they were stored at the museum of the Istituto Policattedra di Oceanologia e Paleoecologia (IPOP) in Catania. All other samples are permanently curated at the MNA and available for study to the scientific community.

## Results

### Taxonomic coverage

**Figure 8.**
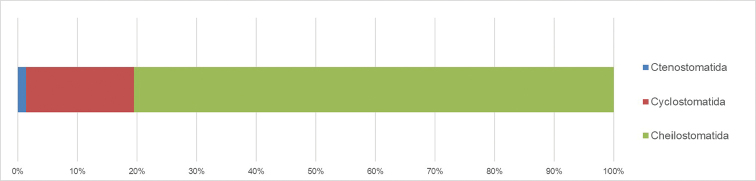
Taxonomic coverage (reported in percentage of specimens per order) of the MNABryozoa collection. Cheilostomatida cover the vast majority of the collection (~80% of the collection specimens), followed by Cyclostomatida (~18%) and Ctenostomatida (~2%).

**Figure 9.**
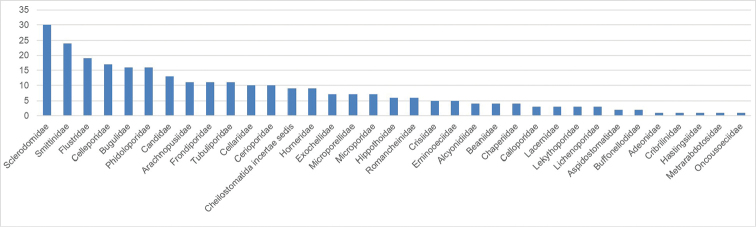
Histogram depicting the number of specimens per family of the MNABryozoa collection.

The present dataset reports distributional data on bryozoan specimens belonging to the classes Stenolaemata and Gymnolaemata collected in the Ross Sea and in Magellan Strait. It includes a total of 311 specimens corresponding to 282 MNA vouchers and belonging to 127 morphospecies. Out of these, 100 were classified to species level and 27 to genus level, representing three orders and 34 families (Figs 8, 9).

**Figure 10.**
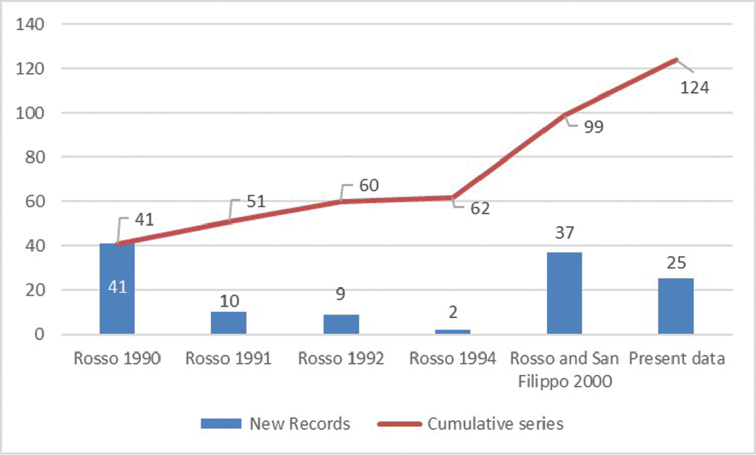
Histogram depicting the number of new records (blue bars) and their cumulative series (red line) reported by literature and this publication for the Terra Nova Bay area.

Considering only the records collected in the Terra Nova Bay area, 41 species (62.12%) were already known for the area, whereas 25 are new records, out of which 23 (34.85%) are classified at species level and two (3.03%) at the genus level. These new records are annotated by ‘NR’ immediately after the species name in the Checklist. By merging previously published records (i.e., Di Geronimo and Rosso 1990, Rosso 1990, Rosso 1991, Rosso 1992a, Rosso 1994, Rosso and Sanfilippo 2000) with the new ones, the number of bryozoan morphospecies occurring in the Terra Nova Bay area increases to 124 (Fig. 10 and Suppl. material 1: Table 1). From this regional checklist we have excluded identifications of specimens lacking key morphological characters, so as to avoid future misidentifications (e.g., Larvaporacf.mawsoni, *Fenestrulina* sp.). Therefore, the total number of species reported in the area may increase in the future.

The MNA collection includes one bryozoan holotype: *Meliceritadigeronimoi* (voucher: MNA 1054). This species, deposited at the museum of the IPOP in Catania (catalogue number IPOP.B1.8.8.1991) in 1991, was published by Rosso in 1992 (Rosso 1992b), i.e., eight years before the establishment of the MNA. To keep track of this voucher, an MNA catalogue number (MNA 1054) has also been assigned, although the sample is curated by the IPOP.

The updated list includes two classes, Stenolaemata, with the order Cyclostomatida (15 morphospecies), and Gymnolaemata, with the orders Ctenostomatida (one morphospecies) and Cheilostomatida (108 morphospecies). The majority of the species are characterized by well-calcified skeletons (88 species), 35 species with slightly calcified skeletons, and only one soft-bodied species (*Alcyonidium* sp., Ctenostomatida). Concerning colony growth habits (i.e., encrusting, erect flexible, erect rigid) (Hageman et al. 1998), erect rigid forms are the commonest (46 species), followed by encrusting (44 species) and erect flexible (32 species) ones.

Old bryozoan names and synonyms were cross-checked in Bryozoa.net (Indexes to bryozoan taxa http://www.bryozoa.net/indexes.html) and WoRMS (World Register of Marine Species; http://www.marinespecies.org; last accessed 20 April 2018). Whenever necessary, taxonomic updates were applied to our checklist. The updated and detailed checklist is reported below.

### Taxonomic update for Cheilostomatida species previously reported in the literature

*Adelascoporajeqolqa* Moyano, 1989 is now *Adelascoporasecunda* Hayward & Thorpe, 1988; *Clithrielluminclusum* (Rogick, 1956), originally classified by Rosso (1990), is now named *Polirhabdotosinclusum* (Waters, 1904) according to the original classification of Hayward and Thorpe; *Cellariavitrimuralis* (Rogick, 1956) (reported by Rosso 1990) is now *Cellariadiversa*, *Celleporellaantarctica* Moyano & Gordon, 1980 (reported in Rosso 1990) is classified as *Antarctothoaantarctica* whereas *Chaperiasimplicissima* Kluge, 1914 is named *Exallozoonsimplicissimum* as stated in Bock and Gordon (2013). Regarding the Family Flustridae, *Flustradrygalskii* Kluge, 1914 (reported by Rosso 1990) is now classified as *Klugeflustradrygalskii* (Bock & Gordon, 2013) (Rosso 1994, Hayward 1995), *Flustraangusta* Kluge, 1914 (reported by Rosso 1990, 1992a, 1994) is *Isosecuriflustraangusta* (Bock & Gordon, 2013) (Hayward 1995), and *Flustratenuis* Kluge, 1914 (reported by Rosso 1994) is *Isosecuriflustratenuis* (Bock & Gordon, 2013) (Hayward 1995). *Hippellozoongelidum* Moyano, 1966 (reported by Rosso 1992) is named *Reteporellagelida* (Waters, 1904) (Hayward 1995, Bock and Gordon 2013). *Mawsoniamembranacea* Livingstone, 1928 (reported by Rosso 1990) is classified as *Swanomiamembranacea* (Thornely, 1924); *Porellaantarctica* Powell, 1967 (reported by Rosso 1991) is *Aimulosiaantarctica* and the genus *Sertella* is now *Reteporella* (Hayward 1995). Among Smittinidae, *Smittinagelida* (as reported by Rosso 1990) is classified as *S.directa* (Waters, 1904) by Hayward (1995), whereas *S.oblongata* (as reported by Rosso 1990), reported as conspecific with *S.antarctica* (Waters, 1904) (Hayward 1995) is classified as *S.antarctica* according to recent taxonomic literature (Bock and Gordon 2013, Kuklinski 2016).

### Taxonomic update for Cyclostomatida species previously reported in literature

*Defranciasarsi* (Borg, 1944), reported for Terra Nova Bay in 2000 (Rosso and Sanfilippo 2000), is here classified as *Apsendesiasarsi* following Bock and Gordon (2013); *Idmidroneamagna* Androsova, 1968 (as reported by Rosso 1990) is synonym of *Idmidroneaobtecta* Borg, 1944. *Tubuliporatubigera* Busk, 1866 (reported by Rosso 1991, Rosso and Sanfilippo 2000) is classified as *Supercytistubigera* as stated in Bock and Gordon (2013). Lastly, although the record *Bearta* sp. has been reported in the literature (Rosso 1990), it is certainly a misspelling and should be referred to *Beania* sp.

### Taxonomic ranks

**Kingdom**: Animalia

**Phylum**: Bryozoa

**Class**: Stenolaemata

**Order**: Cyclostomatida

**Families**: Cerioporidae, Crisiidae, Frondiporidae, Hastingsiidae, Horneridae, Lichenoporidae, Oncousoeciidae, Tubuliporidae

**Genera**: *Bicrisia*, *Crisia*, *Disporella*, *Fasciculipora*, *Hastingsia*, *Hornera*, *Idmidronea*, *Neofungella*, *Oncousoecia*, *Tubulipora*

**Species**: *Bicrisiaedwardsiana* (NR), *Bicrisia* sp., *Crisia* sp., *Disporellahumilis*, *Disporella* sp. (NR), *Fasciculiporaramosa*, *Hastingsiairregularis*, Horneracf.smitti, *Hornerasmitti* (NR), *Hornera* sp., Idmidroneacf.antarctica, Idmidroneacf.obtecta, *Idmidroneaobtecta*, *Idmidronea* sp., *Neofungellaclaviformis*, *Oncousoecia* sp., *Tubulipora* sp.

**Class**: Gymnolaemata

**Orders**: Cheilostomatida, Ctenostomatida

**Families**: Adeonidae, Alcyonidiidae, Arachnopusiidae, Aspidostomatidae, Beaniidae, Buffonellodidae, Bugulidae, Calloporidae, Candidae, Cellariidae, Celleporidae, Chaperiidae, Cribrilinidae, Eminooeciidae, Exochellidae, Flustridae, Hippothoidae, Lacernidae, Lekythoporidae, Metrarabdotosidae, Microporellidae, Microporidae, Phidoloporidae, Romancheinidae, Sclerodomidae, Smittinidae

**Genera**: *Adelascopora*, *Adeonella*, *Aimulosia*, *Alcyonidium*, *Amastigia*, *Amphiblestrum*, *Andreella*, *Antarcticaetos*, *Arachnopusia*, *Austroflustra*, *Beania*, *Bostrychopora*, *Buffonellodes*, *Caberea*, *Camptoplites*, *Carbasea*, *Cellaria*, *Cellarinella*, *Cellarinelloides*, *Celleporella*, *Dakariella*, *Dendroperistoma*, *Ellisina*, *Eminooecia*, *Escharella*, *Exallozoon*, *Exochella*, *Favosthimosia*, *Fenestrulina*, *Flustra*, *Himantozoum*, *Hippothoa*, *Isoschizoporella*, *Isosecuriflustra*, *Klugeflustra*, *Klugella*, *Kymella*, *Lageneschara*, *Larvapora*, *Melicerita*, *Micropora*, *Nematoflustra*, *Notoplites*, *Orthoporidra*, *Osthimosia*, *Pemmatoporella*, *Plesiothoa*, *Polirhabdotos*, *Reteporella*, *Smittina*, *Stephanollona*, *Swanomia*, *Systenopora*, *Thrypticocirrus*, *Toretocheilum*, *Tricellaria*

**Species**: *Adelascoporasecunda*, *Adeonella* sp., *Aimulosiaantarctica*, *Alcyonidiumaustrale*, *Alcyonidium* sp. (NR), *Amastigiacrassimarginata* (NR), *Amphiblestruminermis*, *Andreella* sp., *Antarcticaetosbubeccata*, Arachnopusiacf.aviculifera, *Arachnopusiadecipiens*, *Arachnopusialatiavicularis*, *Arachnopusiamonoceros*, *Arachnopusia* sp., *Austroflustravulgaris*, *Beaniachallengeri* (NR), *Beaniaerecta*, *Beania* sp., *Bostrychoporadentata*, *Buffonellodesumbonata*, *Cabereadarwinii*, *Camptoplitesangustus* (NR), *Camptoplitesbicornis*, *Camptopliteslatus* (NR), *Camptoplites* sp., *Camptoplitestricornis*, *Carbaseacurva*, *Carbaseaovoidea*, *Cellariaaurorae*, Cellariacf.aurorae, Cellarinellacf.latilaminata, Cellarinellacf.nutti, *Cellarinellaedita* (NR), *Cellarinellalatilaminata*, *Cellarinellamargueritae*, *Cellarinellanjegovanae*, *Cellarinellanutti*, *Cellarinellarogickae*, *Cellarinella* sp., *Cellarinellawatersi* (NR), *Cellarinelloidescrassus*, *Celleporella* sp., *Dakariellaconcinna*, *Dendroperistomaprojecta*, *Ellisinaantarctica*, *Eminooeciacarsonae* (NR), *Escharellawatersi*, *Exallozoonsimplicissimum*, *Exochellaavicularis*, *Exochellahymanae*, *Exochellalongirostris*, *Exochella* sp., *Favosthimosiamilleporoides*, *Fenestrulinaparvipora*, *Fenestrulina* sp., *Flustraanguloavicularis* (NR), Himantozoum (Himantozoum) antarcticum, *Hippothoaflagellum*, *Isoschizoporellasecunda* (NR), *Isoschizoporellasimilis*, *Isoschizoporella* sp., *Isosecuriflustraangusta*, *Isosecuriflustra* sp., *Klugeflustraantarctica* (NR), *Klugeflustradrygalskii*, *Klugeflustravanhoeffeni*, *Klugellabuski* (NR), *Kymellapolaris*, *Lagenescharalyrulata*, Larvaporacf.mawsoni, *Larvapora* sp., *Meliceritadigeronimoi*, *Meliceritaobliqua*, *Microporabrevissima*, *Micropora* sp., *Nematoflustraflagellata*, *Notoplitesantarcticus* (NR), *Notoplitesdrygalskii*, *Notoplites* sp., *Notoplitestenuis*, *Orthoporidracompacta* (NR), *Orthoporidra* sp., *Osthimosiabicornis*, *Osthimosiacf.clavata*, *Osthimosiacf.curtioscula*, *Osthimosiaclavata* (NR), *Osthimosiamariae* (NR), *Osthimosia* sp., *Pemmatoporellamarginata*, *Plesiothoacalculosa*, *Polirhabdotosinclusum*, *Reteporellaantarctica* (NR), *Reteporellafrigida*, *Reteporellalongichila* (NR), *Reteporellaparva* (NR), *Reteporella* sp., *Smittinaanecdota* (NR), *Smittinaantarctica*, *Smittinadirecta*, *Smittinapileata* (NR), *Smittinarogickae*, *Smittina* sp., *Stephanollonalongispinata*, *Swanomiabelgica*, *Swanomiamembranacea*, *Systenoporacontracta* (NR), *Thrypticocirruscontortuplicata*, *Thrypticocirrusphylactelloides*, *Toretocheilumturbinatum*, *Tricellaria* sp.

### Spatial coverage of dataset

*General geographic description*:

Ross Sea, Antarctica (Figs 2–4) and the Magellan Strait (Figs 2, 5)

*Coordinates*:

PNRA III expedition: -74.64833 and -74.84833; 164.92167 and 165.61167

PNRA V Exp: -74.69672 and -74.73528; 164.13183 and 164.47500

PNRA VI Exp: -52.51000 and -52.87167; -68.05500 and -74.97500

PNRA XI Exp: -74.69280; 164.60000

PNRA XIII Exp: -74.68850; 164.15833

PNRA XIV Exp: -74.74375; 164.14667

PNRA XVII Exp: -72.65917 and -77.65133; -166.79183 and 176.25783

PNRA XVIII Exp: -74.69557 and -74.79013; 164.03790 and 164.14782

PNRA XIX Exp: -74.82167 and 164.19167

PNRA XXV Exp: -74.69027 and -74.69768; 164.10255 and 164.13108

PNRA XXVII Exp: -74.68562 and -74.71337; 164.05915 and 164.14903

PNRA XXIX Exp: -74.68677 and -74.71828; 164.12278 and 164.24206

*Temporal coverage of dataset*

PNRA III Exp: 5 January – 28 January 1988

PNRA V Exp: 24 December 1989 – 3 January 1990

PNRA VI Exp: 24 February – 3 March 1991

PNRA XI Exp: 22 October 1995 - 2 February 1996

PNRA XIII Exp: 21 February 1998

PNRA XIV Exp: 25 January 1999

PNRA XVII Exp: 4 – 29 January 2002

PNRA XVIII Exp: 31 January – 18 February 2003

PNRA XIX Exp: 20 February 2004

PNRA XXV Exp: 10 December 2009 – 11 January 2010

PNRA XXVII Exp: 28 January - 3 February 2012

PNRA XXIX Exp: 16 January – 1 February 2014

## Description of selected species

**Parent collection identifier**: Italian National Antarctic Museum (MNA, section of Genoa, Italy)

**Collection name**: Bryozoa collection of the Italian National Antarctic Museum (MNA)

**Specimen preservation method**: Part of the old collection was initially fixed in 4% formalin and then transferred in 70% ethanol. Samples collected from 2002 onwards were directly fixed in ethanol (99%) for molecular studies or air-dried. Bryozoan MNA vouchers are now preserved in 90% ethanol (~38% of the entire collection) or dried (~62%).

**Database virtual collection of vouchers and 3D-models**: 3D-models of four Antarctic bryozoans (Figs 11–15) were obtained from four specimens through micro-CT imaging performed at the Department of Geosciences (University of Padua) by CM. Acquisitions were performed using a bench-top Skyscan 1172 micro-CT system (Bruker), equipped with a Hamamatsu 100/250 microfocus X-ray source and a Hamamatsu C9300 11 megapixel camera (with a pixel size of 8.68 μm) filtered by a 0.5 mm Aluminium foil. Projection images were acquired with 70 kV source voltage, 141 μA current, 540 ms exposure time, 2×2 binning mode, 0.25° rotation step over 360°, averaged over 12 frames and in vertical random movement mode to minimise noise, providing an image pixel size of about 9 μm. Two connected scans were necessary to comprise the whole sample height. The run time for each sample was about 400 minutes. Post-acquisition reconstruction was performed using the NRecon (Bruker microCT) software package, starting from raw projection images, and applying thermal correction, misalignment compensation, ring artefact reduction and beam hardening correction. Segmentation was then performed with CT Analyser (Bruker microCT) software package, using a 3D-adaptive thresholding procedure (mean of minimum and maximum value) within spherical kernels of radius 8 pixels, starting from a pre-determined pre-thresholding value. Resulting images were saved as monochrome (1 bit) bitmaps and imported in the CTVox (Bruker microCT) software package to perform 3D-rendering and animations. The model will be available on the MNA web site (www.mna.it) and on Sketchfab (https://sketchfab.com/MNA).

**Figure 11.** Video of the 3D-model of *Hastingsiairregularis* Borg, 1944 (MNA 10490) and *Arachnopusia* sp. (MNA 10491). Height and width of the *H.irregularis* colony are respectively ~5.1 mm and ~6.2 mm.

**Figure 12.** Close-up video of the 3D-model of *Arachnopusia* sp. (MNA 10491) shown in Figure 11. The width of the colony is 1.2 mm.

**Figure 13.** Video of the 3D-model of Idmidroneacf.obtecta Borg, 1944 (MNA 9890). Height and width of the colony are respectively ~20.4 mm and ~17.5 mm.

**Figure 14.** Video of the 3D-model of *Eminooeciacarsonae* (Rogick, 1957) (MNA 8408). Height and width of the colony are respectively ~21.7 mm and ~1.8 mm.

**Figure 15.** Video of the 3D-model of *Smittinadirecta* (Waters, 1904) (MNA 9883). Height and width of the colony are respectively ~17.2 mm and ~16.9 mm.

*Hastingsiairregularis* Borg, 1944 (MNA 10490, Figure 11) belongs to the Cyclostomatida, the only extant order of the class Stenolaemata, whose species are widely spread from all over the world, including the Antarctic Region. The scarce knowledge of cyclostome species for the Ross Sea, compared to cheilostomes, is attributable to the complex taxonomy of the group, which has been studied partly by Borg (1944) and Androsova (1968) but not for the Ross Sea or continent as a whole. The scan shows the structure of an *H.irregularis* colony, which develops well-calcified 3D-architectures, offering a space-resource to other species. *H.irregularis* forms erect colonies composed of ‘fascicles’ of long narrow zooids (ridge-like structures) becoming autozooids at their distal ends. The long and narrow zooids have small-scattered pseudopores and their skeletal walls are characterized by grooves between adjacent zooids and distinct growth ridges. Gonozooids (i.e., reproductive zooids) develops between colony branches (see the median branch developing a gonozooid on the top, Figure 11).

This model clearly provides an example of how the cyclostome colony acts as a resource for another bryozoan genus, *Arachnopusia* (Cheilostomatida, shown in detail in Figure 12). The genus *Arachnopusia* includes encrusting species, usually occurring in shallow shelf seas and only growing on biogenic carbonates (Hayward 1995). The species illustrated in the model (MNA 10491) grows on the reverse (dorsal) side of *H.irregularis* branches, where the surface is smoother compared to the rough substrate created by the tubular processes on the other side. Thus, this encruster might take advantage of the erect habit of the cyclostome (i.e., accessing the food on the water column) without interfering with its activities such as feeding and growing, which mainly occur on the other side.

Idmidroneacf.obtecta Borg, 1944 (MNA 9890, Figure 13) is a cyclostome forming large, erect, well-calcified colonies, with cylindrical branches. These branches are dichotomous, not very regularly spaced, with an oval transverse section, subcircular or rounded trapezoidal. Although bifurcations are not very regular on *I.obtecta* colonies, the whole zoarial shape is 3D-structured. The species represents another example of bioconstructional bryozoan, thus offering substrate and space for other organisms to live and settle. Interestingly, the model shows on reverse sides of branches the presence of arcuate growth lines. The presence of “growth check lines”, especially among Antarctic erect cheilostomes, is very common, indicating a transitional phase between growth and stop in the colony, usually during the winter period (Winston, 1983). These bryozoans represent good bioindicators and key-species for experimental studies thanks to their “growth lines”, which can be easily measured, allowing the quantification of the growth of the entire colony, often related to variations in environmental conditions detectable via stable isotope analyses (i.e., food availability, salinity, temperature and pCO_2_ variations) (Barnes 2015).

*Eminoeciacarsonae* (Rogick, 1957) (NR) (MNA 8408, Figure 14) is an erect calcitic cheilostome bryozoan, originating from an encrusting base. This species develops erect branches, bifurcating dichotomously and shaping 3D colonies. Different characteristic morphological features of the autozooids, such as thick crenulated edges, tuberculate and coarse frontal walls, two pairs of pores at the proximal and distal end of the zooid and frontal suboral avicularia, organized in bands on the colony branches, are illustrated in Figure 14. Being a bioconstructional bryozoan, this species offers space and advantages to other species to live and settle, thus it has a key role in promoting the biodiversity. Described as an endemic Antarctic species, *E.carsonae* is widespread in the Ross Sea (Hayward 1995; Moyano 2005) but it is here recorded for the first time for Terra Nova Bay.

*Smittinadirecta* (Waters, 1904) (MNA 9883, Figure 15), an endemic Antarctic species, represents another example of an erect calcitic cheilostome, developing slender, cylindrical colonies originating from an encrusting base. Its autozooids, budding simultaneously, are organized in whorls of four or five, with frontal orifices opening around the whole branch (Figure 15). The primary orifice has an anvil-shaped lyrula (e.g., median tooth) with a straight edge projecting corners and a peristome, and its distal third is in a continuum with the calcification of the next autozooid (Hayward 1995). *Smittinadirecta* is subject to marked secondary calcification processes, which lead, in later ontogeny, to the thickening of the frontal walls, thus some of its morphological features are hardly recognizable. In addition to the autozooids, the branch represented in Figure 15 bears kenozooids (e.g., zooids without muscles and primary orifice), whose main function is to provide structural strength to the colony.

## Datasets

*Dataset metadata*:

This dataset contains data on the phylum Bryozoa, represented by two classes and three orders in the Ross Sea, with focus on Terra Nova Bay, and the Magellan Strait. The present dataset has been formatted in order to fulfil the Darwin Core standard protocol required by the OBIS scheme (http://www.iobis.org/manual/lifewatchqc/) and according to the SCAR-MarBIN Data Toolkit (available at http://www.scarmarbin.be/documents/SM-FATv1.zip). The dataset was uploaded and integrated with the ANTOBIS database (the geospatial component of SCAR-MarBIN). Two studies have been based on this dataset: Rosso and Sanfilippo 1991; Rosso 1992b.

The Darwin Core elements included in the dataset are: ID, Institution code (i.e., the name of the institution where the samples are curated), basis of record, occurrence ID, catalogue number (i.e., MNA catalogue number), individual count, preparation (preservation method and more info about the sample, e.g., ETOH, dry, glass slides, etc.), event ID (i.e., original sampling station code), sampling protocol (sampling gear), event date, year, month, day, verbatim event date, field number (sampling station code as showed in the maps), event remarks (i.e., expedition), maximum depth meters, decimal latitude, decimal longitude, locality, taxon ID, scientific name ID, scientific name, kingdom, phylum, class, order, family, genus, subgenus, specific epithet, infraspecific epithet, scientific name authorship, taxon remarks. Some of the sampling stations are dredge stations, which have two sets of coordinates: the starting and ending points. In such cases, the coordinates reported in the dataset refer to the starting point of the dredge station.

**Object name**: Bryozoa collection of the Italian National Antarctic Museum (MNA) – Data

**Character encoding**: UTF-8

**Format name**: Darwin Core Archive format

**Format version**: 1.0

**Distribution**: https://doi.org/10.15468/u08az1

**Language**: English

**Metadata language**: English

**License of use**: This dataset [Bryozoa collection of the Italian National Antarctic Museum (MNA) - Data] is made available under the Creative Commons Attribution License (CC-BY) 4.0: http://www.creativecommons.org/licenses/by/4.0/legalcode

**Date of metadata creation**: 25 May 2018

**Hierarchy level**: Dataset

## Supplementary materials

Supplementary material 1 Terra Nova Bay check-list of Bryozoa species Data type: (measurement/occurrence/multimedia/etc.) This is an open access article distributed under the terms of the Creative Commons Attribution License (CC BY 4.0), which permits unrestricted use, distribution, and reproduction in any medium, provided the original author and source are credited.Matteo Cecchetto, Chiara Lombardi, Simonepietro Canese, Silvia Cocito, Piotr Kuklinski, Claudio Mazzoli, Stefano Schiaparelli
